# Modeling correlated information change: from conditional beliefs to quantum conditionals

**DOI:** 10.1007/s00500-017-2499-5

**Published:** 2017-02-25

**Authors:** Alexandru Baltag, Sonja Smets

**Affiliations:** ILLC, University of Amsterdam, Amsterdam, The Netherlands

**Keywords:** Belief Revision, Test Frame, Conditional Belief, Propositional Dynamic Logic, Dynamic Epistemic Logic

## Abstract

In this paper, we propose a unified logical framework for representing and analyzing various forms of correlated information change. Our main thesis is that “logical dynamics,” in the sense of van Benthem (Exploring logical dynamics. CSLI Publications, Stanford, 1996; Logical dynamics of information and interaction. Cambridge University Press, Cambridge, 2011), and in particular dynamic epistemic notions of conditional, as developed in Baltag and Smets (Electron Notes Theor Comput Sci 165:5–21, 2006a; Stud Log 89:185–209, 2008a; Texts in logic and games. Amsterdam University Press, Amsterdam, pp 9–58, 2008b), play a central role in understanding and modeling a wide range of apparently very different information-gathering phenomena which do have one specific feature in common, namely the very act of learning new information may directly change the reality that is being learned. On the one hand, we focus on the way in which an introspective agent changes her beliefs when learning new higher-order information, i.e., information that may refer to her own beliefs. On the other hand, we analyze situations in which an observer learns about a phenomenon by performing observations that may perturb the very phenomenon under study, as in the case of quantum measurements, or observations in social sciences, psychology and medicine. Our formal techniques are based on ideas from dynamic logic and on the modeling of “dynamic conditionals.” We offer a semantics based on “test frames,” i.e., Kripke frames labeled by propositional formulae which yields a unified setting for the two types of correlated information change under study. We show how this framework can be used to analyze the ontic and epistemic–informational aspects of quantum measurements and to compare them with other types of observation, testing, belief revision, counterfactual conditionals, etc.

## Introduction

In this paper, we focus on modeling situations that exhibit a particular form of “correlated information change,” i.e., situations in which the very act of learning new information may influence the result and changes the very phenomenon under study. Examples of correlated information change appear in a number of different areas ranging from quantum mechanics, to logic, medical science and social science. In these areas, different forms of correlated information change have been identified and studied. The quantum observer effect, embodied in the motto that “observation causes perturbation,” lies at the basis of contemporary applications of quantum mechanics to secure communication. The Hawthorne effect (Levitt and List 2009) in social science points to the experimenter’s influence on the agent’s social or behavioral attitudes under study. The placebo effect in medical science indicates the effect of a non-active substance given to medical patients. But philosophers and logicians noticed already long time ago that an analog of the observer effect occurs whenever a rational introspective agent has to revise her beliefs with higher-order information, i.e., information that refers directly to the (prior or posterior) beliefs of the very same agent. A specific example is given by belief revision with a Moore sentence (Moore 1942). We will come back to this specific example later on, for now it is important to note what all these examples have in common: namely that a true (respectively, false) property can become false (respectively, true) by the very act of observing, learning, communicating or simply accepting (the truth of) that property. To deal with this range of different information-gathering phenomena, coming from areas as diverse as quantum physics and the social sciences, a unified logical setting is required.

In this paper, we zoom in on two leading examples: the first comes from the area of belief revision theory and the second deals with quantum observations. The common formal setting that can subsume the different approaches of belief revision and quantum observations is given by what we call “test frames,” these are labeled Kripke frames in which the basic relations $R^{P}$ are labeled by “propositions” (subsets *P* of the state space). Such structures are special cases of so-called dynamic frames and are well studied in the context of propositional dynamic logic (PDL) (Harel et al. 2000). More generally, such labeled relations can receive different interpretations, depending on the context in which they are used. In this paper, we show that they can be used to model various notions of conditionals ranging from those based on classical “test” actions; quantum “test” actions; counterfactual conditionals encoding reasoning about hypothetical situations; doxastic conditionals that internalize such hypothetical reasoning in the form of conditional beliefs, or knowledge about counterfactuals; and finally various forms of knowledge update or belief revision that capture the information change induced by (classical or non-classical) observations. Having a unified semantics facilitates the comparison of the different notions of conditionals that we offered in Baltag and Smets (2008a). This shows the fruitfulness of a unified approach for analyzing and classifying various forms of information flow, in terms of their underlying relational structure.

Sections 2 and 3 contain background material, summarizing relevant concepts and results from the literature (including some due to our own previous work in e.g., Baltag and Smets (2005, 2006a, b, 2008a, b)), though presenting them from a new perspective. In Sect. 2, we focus on the first type of scenarios under investigation, and these come from the area of belief revision theory. Within the general context of focussing on “logical dynamics” (in the sense of van Benthem 1996, 2011), we first provide the necessary background information about AGM belief revision theory before introducing the formal notion of a test frame and explaining the conditions that are needed in order to use this setting to model belief revision scenarios. In Sect. 3, we focus on the second type of scenarios under investigation, and these come from the area of quantum mechanics. After providing the necessary background information about the logical foundations of quantum mechanics, we present some of our own past work on quantum dynamic logic, based on quantum test frames. In Sect. 4, we generalize this work, by introducing the new concept of general test frames and investigating their properties and the corresponding logic. In Sect. 5, we show how our setting subsumes the various forms of conditionals and learning mentioned above and show that in the quantum case our framework leads to an epistemic extension of quantum dynamic logic, obtained by making explicit the observer’s information state and observations. In Sect. 6, we use this unified framework to compare the properties of these various types of conditionals and updates. We end with some concluding remarks about the erosion of the distinction between “ontic” and “epistemic” in the quantum realm, arguing that far from reducing quantum behavior to a purely informational phenomenon, this shows that on the contrary knowledge and information gathering are “real” physical phenomena, with real consequences in the actual world.

## Belief revision with higher-order information

The so-called AGM theory of belief revision originated with the work of Alchourrón et al. (1985). The AGM theory provides a syntactic setting to represent the agent’s basic beliefs and their belief changes. AGM represents the basic beliefs of an agent by a theory *T*, i.e., a consistent set of sentences coming from a given propositional language *L*. The set *T* is taken to be closed under logical consequence and can be subject to revision when the agent faces new incoming information that affects the agent’s basic beliefs. Take for instance the following example:

**Example: Basic belief revision.** Let *T* be the theory representing the current beliefs of a given agent who we call Alice. Every sentence that is contained in Alice’s theory *T* is a sentence that she accepts. Now let $\lnot p$ be a sentence stating that the lottery ticket in Alice’s hand is not the winning one. We assume that $\lnot p$ is included in *T*, i.e., $\lnot p \in T$, which indicates that Alice currently believes $\lnot p$. Consider now that our agent hears the truthful announcement of the fact that *p*, indicating that she holds the winning lottery ticket. As a consequence of this announcement, Alice will revise her beliefs and come to believe that she has won, i.e., $T * p$ represents the new theory *T* revised by the fact that *p*. Note that the new incoming information *p* directly contradicts the prior belief of Alice about $\lnot p$. In order to reach a consistent theory *T*, Alice has to give up her belief in $\lnot p$ and replace it with a belief in *p*.

In order to compute Alice’s revised beliefs, the AGM belief revision theory uses a revision operator $*$. The $*$ revision operator takes as input both a prior belief set *T* and a given sentence $\varphi$ from the language *L* in order to yield a revised belief set $T * \varphi$. The operation $*$ is subject to a number of axioms or so-called postulates which are supposed to encode the principles of rational belief revision. Without going into the full details on the AGM revision postulates (Alchourrón et al. 1985), we highlight here just one of them, i.e., the success postulate. As we show further on, the success postulate is especially important in the context of belief revision with higher-order information.

The AGM success postulate states that the target formula $\varphi$ (the new incoming information) is always contained in the revised theory, i.e., $\varphi \in T * \varphi$. Similar to some of the other AGM postulates, which have been debated in the philosophical literature, also the success postulate can be contested as a principle of rationality. The success postulate, when we read it as “the new incoming information is accepted or believed after the act of learning it,” imposes an inherent limitation on the type of belief revision that can be performed. In particular, under this specific reading of the success postulate, AGM revision does not take into account that the very fact of learning new information itself can disturb the truth value of what is being learned. So while overall the AGM theory is fit to model the above belief revision example 1 of our agent who learns that she holds a winning lottery ticket, there are many scenarios involving belief revision with higher-order information which on first sight seem to fall outside the scope of the AGM theory. These are exactly the scenarios which involve the correlated information change that we mentioned in the previous section.

### Belief revision with higher-order information

Let us start with another example:

**Belief Revision with a Moore sentence** We assume that our agent Alice holds certain believes which are represented by a theory of beliefs *T*. Again we let $\lnot p$ be included in *T*, which indicates that Alice currently believes that she does not hold the winning lottery ticket. Assume next that Alice receives a piece of new incoming information $\varphi$ which represents the following true sentence: “you have won the lottery but you don’t believe it” (formally this can be modeled as $p \wedge \lnot Bp$). Upon hearing $\varphi$, Alice tries to learn it. $\varphi$ is a conjunction and hence Alice will need to learn both conjuncts. So after learning the conjunct *p*, Alice accepts that *p* is the case and as a direct consequence she no longer believes $\lnot p$. If Alice is an introspective agent, then not only she will have come to accept *p* but she also accepts *Bp*. Yet at this point, after accepting the first conjunct *p* of $\varphi$, Alice cannot accept the second conjunct of $\varphi$ while keeping her beliefs consistent.

The sentence $\varphi$ is a typical example of a Moore sentence. The very act of trying to learn this Moore sentence will change its truth value. Note that $\varphi$ is true before anyone tells $\varphi$ to our agent, but it is no longer true after she has learned it. This example gives an illustration of a correlated information change, and it shows that there are “truths” that cannot be known/believed nor learned.

In conclusion, if we extend the AGM setting to this case of belief revision with higher-order doxastic or epistemic information (as in the above example), we observe that the original reading of the AGM success postulate can no longer be sustained. Indeed, the case of revision with a Moore sentence, the success postulate (under the above given reading) would force us (as a principle of rationality) to acquire false beliefs!

Such examples show that there is a problem with accepting the success postulate (in its original reading) in the context of revision with higher-order information. In essence, there are at least two solutions to this problem. The first solution is to accept that the AGM theory only works with a restricted language *L*, i.e., a setting in which theories *T* can contain the basic sentences (and their Boolean combinations) of a given propositional language but should not contain sentences having modalities that refer to the beliefs of agents. This solution boils down to a belief revision setting that cannot model higher-order reasoning nor any type of revision with sentences that explicitly refer to the beliefs of agents. While this first solution solves the problem on technical grounds, it is not satisfactory as real agents *can* indirectly learn new facts about the world via higher-order sentences. The second solution we propose focuses on another way to model belief revision. In this second solution, we are still validating the AGM postulates, but we reinterpret them in such a way that they can be made plausible in the context of revision with higher-order information. The latter solution is the one we adopt in the remainder of this section and to explain it we will make use of the tools of dynamic logic.

### Models for belief revision

Let us start by offering a new interpretation to $T * \varphi$, the “revised beliefs” of an agent. We will treat $T * \varphi$ as being the beliefs (held after learning $\varphi$) about *the same state* as the unrevised beliefs (i.e., the state before the announcement of $\varphi$). As we work from now on in a modal logic setting, we are replacing the above $*$-notation with the conditional belief notation $B^{\psi }{\varphi }$, which we read as “the (implicit) agent believes $\varphi$ conditional on $\psi$.” Intuitively, this means that, in case the agent will find out that $\psi$ was the case, she will believe that $\varphi$ *was* the case (*before* the learning act) (Baltag and Smets 2008b). We can also use $K\varphi$ to express the fact that *the agent knows* $\varphi$ (with absolute certainty).

So the language $\mathcal{L_B}$ that we work with is defined by recursion in BNF format:

$$\begin{aligned} \varphi {::=} \bot \, | \, p | \, \varphi \rightarrow \varphi \, | \, K\varphi \, | \, B^{\varphi }{\varphi } \end{aligned}$$

In the language $\mathcal{L_B}$, we define the classical negation $\lnot \varphi \,{:=}\,\varphi \rightarrow \bot$, tautology $\top \,{:=}\,\lnot \bot$, classical disjunction $\varphi \vee \psi$ and classical conjunction $\varphi \wedge \psi$ as standard. The agent’s *default (non-conditional) beliefs* are given by putting $B \varphi \, {:=}\,B^{\top }{\varphi }$. (In fact, we will show that in this setting knowledge *K* can also be defined in terms of conditional beliefs, but we chose here to take it as a primitive operator.)

Using the language $\mathcal{L_B}$, we can express the success postulate as follows:

$$\begin{aligned} \vdash B^{\varphi } \varphi \end{aligned}$$

Only when reading the act of belief revision in this “static” way, as talking about an unchanging world, we can make sense of the AGM postulates. This stands in contrast to the idea of “dynamic belief change,” which keeps track of both the epistemic and ontic changes in a changing world, but it does so in a setting in which the AGM postulates cannot be maintained for the dynamic belief change operator. While this difference between static AGM revision and dynamic belief change is further explained in Baltag and Smets (2008b), we note here only that in order to model true belief dynamics the logical language will have to be extended. The required extension includes an “update” operator $[\psi !]\psi$ in the style of Public Announcement Logic (Plaza 1989). The expression $[\psi !] B \varphi$ to encode that “after learning $\psi$, the agent believes $\varphi$ (in the state of affairs *after* the learning act).” In this extended setting, the success postulate fails for non-atomic sentences $\varphi$ (for instance, in the case of Moore sentences)

$$\begin{aligned} \not \vdash [\varphi !] B \varphi , \end{aligned}$$

though it is valid for atomic formulas

$$\begin{aligned} \vdash [p!] B p, \end{aligned}$$

and in fact we have the stronger validity

$$\begin{aligned} \vdash [p!] K p. \end{aligned}$$

The standard semantics for belief revision is due to A. Grove, who adapted D. Lewis’ sphere models (for reasoning about counterfactuals) to fit it in line with doxastic conditionals. Grove models have an equivalent *relational* presentation, which we adopt here:

**Definition** *[Plausibility Frames and Models]* A *(single-agent) plausibility frame* is a structure $(S, \le )$, consisting of a set *S* of states and a binary “(im)-plausibility” relation $\le \subseteq S\times S$. We read $s\le t$ as “state *s* is at least as plausible as state *t*.” So, for any given set $P\subseteq S$, the “minimal” *P*-states in the set $Min_{\le } P{:=}\{s\in P: s \le t \hbox { for every } t\in P\}$ represent *the most plausible* *P*-states. The plausibility relation $\le$ is required to be a total, well-founded preorder^1^ on *S*. A *plausibility model* $(S, \le , \Vert \bullet \Vert )$ consists of a plausibility frame $(S, \le )$, together with a valuation map assigning to each atomic formula $p\in \Omega$ some subset $\Vert p\Vert \subseteq S$.

**Semantics for Belief Revision** The semantics is given in the usual way, by defining a satisfaction relation $s\models \varphi$ between sentences and formulas, or equivalently an interpretation map $\Vert \varphi \Vert {:=}\{s\in S: s\models \varphi \}\subseteq S$ for each formula. The semantics is standard for atomic sentences, $\bot$ and classical implication, while for the others we put:

$$\begin{aligned}&s \models B^{\varphi } \psi \quad \hbox { iff } Min_{\le } \Vert \varphi \Vert \subseteq \Vert \psi \Vert \\&s\models K\varphi \qquad \hbox {iff } S\subseteq \Vert \varphi \Vert \end{aligned}$$

In other words, $\psi$ is believed given $\varphi$ whether it is true in all the most plausible $\varphi$-states, while $\varphi$ is known whether it is true in all states. It is easy to see that $K\varphi$ is equivalent to $B^{\lnot \varphi }\bot$ (so that *K* is in fact definable in terms of conditional belief).

The usual way to extend this semantics to dynamic update operators $[\varphi !]\psi$ is to consider these a model-changing operator. But in the single-agent case, this is equivalent to a simpler presentation, in the spirit of the Subspace Set Logic (or topo-logic) semantics, as operators that change the agent’s “information state”: for each possible “information states” $T\subseteq S$, define a *T*-*interpretation* $\Vert \varphi \Vert _T$ of every formula $\varphi$; equivalently, define a satisfaction relation $(s,T)\models \varphi$ between *pairs* (*s*, *T*) of an “ontic state” $s\in S$ and an “information state” $T\subseteq S$ with $s\in T$, on the one hand, and formulas $\varphi$, on the other hand. (We can go back and forth between these two notions, by putting $(s,T)\models \varphi$ iff $s\in \Vert \varphi \Vert _T$, and conversely putting the $\Vert \varphi \Vert _T{:=}\{s\in T: (s, T)\models \varphi \}$.) The full semantics is given by putting

$$\begin{aligned}&\Vert \bot \Vert _T=\emptyset ,\\&\Vert p\Vert _T{:=} T\cap \Vert p\Vert \hbox { (where }\Vert p\Vert \hbox { is the valuation )}\\&\Vert \varphi \rightarrow \psi \Vert _T= \{s\in T: \hbox { if } s\in \Vert \varphi \Vert _T \hbox { then } s\in \Vert \psi \Vert _T\},\\&\Vert B^{\varphi }\psi \Vert _T=\{s\in T: Min_{\le } \Vert \varphi \Vert _T\subseteq \Vert \psi \Vert _T\},\\&\Vert K\varphi \Vert :=\{s\in T: T\subseteq \Vert \varphi \Vert _T\},\\&\Vert [\varphi !]\psi \Vert _T=\{s\in T: \hbox { if } s\in \Vert \varphi \Vert _T \hbox { then } s\in \Vert \psi \Vert _{\Vert \varphi \Vert _T}\} \end{aligned}$$

The resulting Conditional Doxastic Logic with updates (*CDL*!) allows us to capture the mentioned scenarios of belief revision with higher-order information. A complete axiomatization of this logic is in our paper (Baltag and Smets 2008b).

## Quantum observer effect

An area of research where correlated information change has been effectively studied, using sophisticated mathematical techniques, is quantum mechanics. The quantum observer effect can be viewed as a special case of correlated information change between two physical systems: the observed system and the observer. Every act that an observer performs in order to extract information about a quantum system may have ontic side effects. If we follow the standard interpretation of quantum mechanics, a system in a superposition state can collapse into an eigenstate when it is being measured. This indicates that observations, or epistemic acts of information extraction, can induce a change in the ontic state of a quantum system. In Baltag and Smets (2008a), we expressed it via the following slogan: in a quantum universe there is no information change without changing the world. Moreover, in the case of quantum observations we deal with an increased uncertainty when measuring incompatible physical properties. Certain physical properties such as position and momentum are incompatible with each other, which means that both cannot be measured with arbitrary precision. Formally such properties are represented by non-commuting observables. After consecutively testing two such incompatible properties, the acquired information about the first property gets overwritten by testing for the second one. This last feature explains the non-monotonic dynamics of quantum information change due to measurements. These non-classical effects together indicate that the way how quantum information changes under observations should play a crucial element in our understanding of the nature of quantum information. Yet traditional quantum logic has designed models for the representation of quantum information that do not take these dynamic ingredients into account. We argue below that a semantics based on test frames can replace the traditional static view and provide us with a better account to model quantum observations.

### Traditional quantum logic

As usually presented, quantum logic (QL) is a non-classical logic with the same syntax as standard propositional logic, but with different laws. We start from the Backus–Naur Form (BNF) of the following language $L_Q$ which we use to talk about the physical properties of a given quantum system. The language $L_Q$ is build up from a given set $\Omega$ of atomic propositions *p*:

1 $$\begin{aligned} \begin{array}{llllllll} \varphi \, \, \, {::=} \, \, p&\mid \, \, \varphi \wedge \varphi&\mid \, \, \sim \varphi \end{array} \end{aligned}$$

This BNF definition specifies that the well-formed formulae of $L_Q$ comprise only those symbols generated recursively from the atomic propositions, the conjunction $\wedge$ and orthocomplement (or quantum negation) $\sim$. In this language, the quantum disjunction can be defined as the De Morgan dual under the quantum negation of the conjunction:

$$\begin{aligned} {\varphi \sqcup \psi }\,{:=}\,{\sim (\sim \varphi \wedge \sim \psi )} \end{aligned}$$

The best candidate for a quantum conditional is the so-called Sasaki Hook, which is definable in terms of the other operators as follows:

$$\begin{aligned} \varphi \rightsquigarrow \psi \, \,: = \, \, \sim \varphi \sqcup (\varphi \wedge \psi ) \end{aligned}$$

We define *falsum* as $\bot {:=} p \, \wedge \sim p$ and *verum* as $\top {:=} \sim \bot$.

**Hilbert Space Semantics** The standard semantics for quantum logic is given in terms of the Hilbert space formalism of quantum mechanics. The *state* of a physical system is represented by a “ray” (or a one-dimensional subspace) of a given Hilbert space *H*. The possible *physical properties* (or propositions) of the system correspond to *closed linear subspaces* of *H*. A state *satisfies* a property if it belongs to the corresponding subspace. The sentences of $Q_L$ are hence interpreted as linear subspaces. The orthocomplement $\sim \varphi$ is the *orthogonal subspace to* $\varphi$. The conjunction $\varphi \wedge \psi$ corresponds to the *intersection* of the two subspaces. The quantum disjunction $\varphi \sqcup \psi$ represents the *subspace generated by the union* of the two subspaces represented by $\varphi$ and $\psi$.

In the history and development of ortho(modular)quantum logic, we encounter several kinds of axiomatizations ranging from natural deduction systems to Hilbert-style axiomatizations and Gentzen-style sequent calculi. We do not review these systems here, but note that such proof systems have been designed to provide the axioms and/or rules that regulate the operators of the language $L_Q$ according to the laws of quantum theory. An essential feature of these systems is that the distributivity of the conjunction and quantum disjunction cannot be maintained and is replaced by a weaker so-called orthomodular law. Against the background of these systems, one can then highlight how the quantum disjunction, quantum negation and quantum implication differ from their classical counterparts.

Superpositions of physical properties are captured in $L_Q$ by using the quantum disjunction $\sqcup$, which differs from the classical disjunction. Indeed, a physical system in a superposition state *s*, which is taken to be the superposition of two states $s'$ and $s''$ where $s'$ satisfies $\varphi$ and $s''$ satisfies $\psi$, can fail to satisfy either $\varphi$ or $\psi$. Hence, contrary to the classical disjunction, the superposition of two properties $\varphi$ and $\psi$ can be true in a state *s*, while both $\varphi$ and $\psi$ are false in state *s*:

$$\begin{aligned}&s \, \in \, \Vert \varphi \Vert \, \mathrm { or } \,\, s \, \in \, \Vert \psi \Vert \,\, \Rightarrow \, s \, \in \, \Vert \varphi \, \sqcup \, \psi \Vert \\&\qquad \hbox { (but the converse fails) } \end{aligned}$$

The behavior of the orthocomplement, expressed using $\sim$, also differs from the classical negation. A system in state *s* may well fail to satisfy $\varphi$ without being in a state in which the orthocomplement of $\varphi$, i.e., $\sim \varphi$, is true:

$$\begin{aligned} s \, \in \, \Vert \sim \varphi \Vert \, \Rightarrow \, \, s\, \not \in \, \Vert \varphi \Vert \qquad \hbox {(but the converse fails)} \end{aligned}$$

Besides the quantum disjunction and negation, quantum logicians have studied the formal properties of the quantum implication (see, e.g., Kalmbach 1983; Hardegree 1975; Marsen et al. 1981; Hardegree 1979; Coecke and Smets 2004; Smets 2001). Among the different candidates for a nice quantum implication that one can consider, it is important to note that the above Sasaki Hook has some similarities to the material implication of classical logic. In particular, the Sasaki Hook behaves *locally Boolean*, which means that if we restrict the logic to its Boolean fragment then the Sasaki Hook reduces to the classical material implication. In general, however, the classical deduction theorem does fail for it and that result goes hand in hand with the mentioned failure of distributivity of the conjunction and disjunction.

While it is interesting to compare the behavior of the mentioned quantum operators to their classical counterparts, a clear intuitive physical explanation remains a point of debate. This quest for a physical explanation of the quantum logical principles and laws goes back to Birkhoff and von Neumann who ended their 1936 paper (Birkhoff and Neumann 1936) exactly with the question what a simple and plausible physical motivation for the modular law would be. What we show below is that if we reinterpret traditional quantum logic in a framework suitable for dynamic logic, we are getting much closer to providing a more plausible physical motivation.

### Dynamic quantum logic

In Baltag and Smets (2005), we have launched the idea of how Hilbert spaces can be structured as non-classical relational models, making it possible to use the methods and tools of modal logic to model quantum information. These models are based on what we called *quantum test frames* of the form

$$\begin{aligned} (S, \{\mathop {\rightarrow }\limits ^{P}\}_{{ P} \in \mathcal{L}}), \end{aligned}$$

where the elements of *S* are taken to represent the possible ontic states of a given physical system. The labeled accessibility relations in the quantum test frame describe the changes of state induced by *possible actions* that may be performed on the system. The accessibility relations are labeled by “test” actions *P*?, where the labels for the tests come from a given family $\mathcal{L}\subseteq \mathcal{P}(S)$ of subsets $P\subseteq S$, which are called the testable properties. These tests are meant to represent successful part of a yes–no measurement of a physical property. So test *P*? indicates that property *P* is successfully tested: it means the outcome is positive and as a result of this the state of the (observed) system collapses to a state satisfying property *P*.

**Definition** [*Quantum Test Frames*]. Any Hilbert space *H* can be structured as a quantum test frame, by taking as set of states *S* the set of all “rays” (one-dimensional subspaces) of *H*, taking the family ${\mathcal L}$ of testable properties to consist of all (subsets of *S* corresponding to) closed linear subspaces *P* of *H*, and taking $\mathop {\rightarrow }\limits ^{P}$ to be the (partial function induced on *S* by) the *projector* $Pr_P$ onto the (subset of *S* corresponding to) the linear subspace *P*.

The logical language for quantum observations $L_O$ can then be defined as follows:

$$\begin{aligned} \begin{array}{ccc ccc ccc ccc cc c} \varphi&{:: =}&p |&\varphi \wedge \varphi |&[\varphi ?]\varphi \end{array} \end{aligned}$$

The variables *p* comes from a given set of basic (elementary) propositions $\Omega$. In this language, $\varphi ?$ denotes the quantum test action and $[\varphi ?]\psi$ is a binary operator which takes as input a test action $\varphi ?$ and a formula $\psi$. If we define the quantum negation as $(\sim \varphi ) := [(\varphi )?]\bot$, we can introduce the quantum disjunction as the De Morgan dual of the conjunction $\varphi \sqcup \psi {:=} \sim (\sim \varphi \wedge \sim \psi )$.

Our models are obtained by extending quantum test frames with a valuation map $\Vert \bullet \Vert$ for atomic formulas (similar to the valuation in plausibility models). The semantics is given by extending the valuation map to an *interpretation* $\Vert \varphi \Vert \in \mathcal{L}$ for *all* formulas $\varphi$ of $L_O$, while quantum tests $\varphi ?$ are interpreted using the transition relations labeled by $\Vert \varphi \Vert ?$. We use the weakest precondition to give an interpretation to the labeled modal operator:

$$\begin{aligned} \Vert [\varphi ?] \varphi \Vert =\{ s\in S: \forall t (s\mathop {\rightarrow }\limits ^{\varphi ?} t \Rightarrow t\in \Vert \varphi \Vert ) \} \end{aligned}$$

In this logical setting, the *orthocomplement* ${\sim \varphi }$ of a property explicitly expresses the *impossibility of a successful test* of $\varphi$. Moreover, the Sasaki Hook can be defined as the weakest precondition of a quantum test action: $\varphi \rightsquigarrow \psi {:=} [\varphi ?] \psi$. In other words, we obtain a dynamic relational interpretation of the non-classical connectives of quantum logic. In Baltag and Smets (2005), we extended the above-mentioned quantum test frames to what we called quantum dynamic frames by adding a second type of relation, labeled by quantum “actions” $a \in A$ given by so-called *unitary evolutions* (quantum gates):

$$\begin{aligned} \left( S, \{\mathop {\rightarrow }\limits ^{P?}\}_{P \in \mathcal{L}}, \{ \mathop {\rightarrow }\limits ^{a} \}_{a \in {A}} \right) \end{aligned}$$

In essence, these dynamic frames distinguish two different types of actions that a system can be subjected to, namely quantum tests which are used to model observations in which agents interact with the system and unitary evolutions in which we let the system evolve but no interaction with the observer takes place. These dynamic frames are fit to fully model quantum systems when we impose a set of ten abstract semantic constraints on the labeled relations which correspond to a number of axioms in the proof system. This setting allows us to express all the important qualitative properties of single quantum systems as dynamic-logical properties. In particular, some unnatural postulates of quantum logic of a rather technical nature, such as the mentioned law of weak modularity, can be recovered as natural (although non-classical) properties of the quantum logical dynamics. In Baltag and Smets (2005), we prove an “abstract completeness result” for the axiomatic proof theory, showing that all qualitative features of single quantum systems are captured by our axioms. In our work (Baltag and Smets 2006b), we added (qualitative) spatial features to quantum dynamic logic, allowing us to talk about local properties of given subsystems of a quantum system. This made possible an analysis of the dynamic–informational aspects of compound systems. We showed that notions such as entanglement and Bell states can be given a logical characterization, and we used this logic to study multi-partite quantum information flow.

## General test frames

We proceed now to generalize quantum test frames to obtain the new notion of *general test frames*. These provide a uniform framework for representing *conditionals* of various kinds (classical, quantum, counterfactual, doxastic, epistemic or dynamic conditionals), as special case of weakest preconditions of test actions.

For any binary relation $R\subseteq S\times S$ on a given set *S*, and any subset $P\subseteq S$, we use the following notations:

$$\begin{aligned}&R[P]\,\, {:=}\,\, \{y\in S: \exists x\in S\, (xRy \wedge x\in P)\},\\&[R]P\,\, {:=}\,\, \{x\in S: \forall y\in S\, (xRy \Rightarrow y\in P)\}. \end{aligned}$$

Note that [*R*]*P* corresponds to the standard Kripke (semantics for the) modality associated with the accessibility relation *R*. As well known in Computer Science, when *R* is interpreted as an “action” or a “program,” *R*[*P*] represents *the strongest postcondition* ensured (on the output) by (doing action) *R* on *P*-inputs, while [*R*]*P* represents* the weakest precondition* (on the inputs of *R*) ensuring that *P* will be satisfied on the output after (doing action) *R*. In particular, when $P=\{s\}$ is a singleton with $s\in S$, we put

$$\begin{aligned}&R[s] \,\, {:=}\,\, R[\{s\}]= \{y\in S: sRy\},\\&[R]s \,\, {:=}\,\, [R]\{s\}= \{x\in S: \forall y\in S (xRy \Rightarrow y=s)\}. \end{aligned}$$

**Definition** [*General Test Frames*]. A *general test frame* (or “test frame,” for short) is a relational Kripke frame

$$\begin{aligned} (S, \{\mathop {\rightarrow }\limits ^{P}\}_{{ P} \in {\mathcal L}}), \end{aligned}$$

consisting of a set *S* of *states*, a family ${\mathcal L}\subseteq {\mathcal P}(S)$ of *testable propositions* (or “theories,” or “information states”), and a family of binary (accessibility) $\mathop {\rightarrow }\limits ^{P} \subseteq S \times S$ labeled by testable propositions $P\in {\mathcal L}$, assumed to satisfy the following five conditions:

0. (**Closure**) *The family* ${\mathcal L}$ *of testable propositions contains the inconsistent proposition and the tautological one and is closed under arbitrary intersections, weakest preconditions and strongest postconditions*: i.e., $\emptyset , S\in {\mathcal L}$; if $\{P_i:i\in I\}\subseteq {\mathcal L}$ then $\bigcap _{i\in I} P_i\in {\mathcal L}$; and, if $P,Q\in {\mathcal L}$, then $\mathop {\rightarrow }\limits ^{P}[Q]\in {\mathcal L}$ and $[\mathop {\rightarrow }\limits ^{P}]Q\in {\mathcal L}$.
1. (**Testability**) *If a testable proposition is true, then it can be successfully tested*: i.e., if $s \in P\in {\mathcal L}$, then there exists some state *t* with $s \mathop {\rightarrow }\limits ^{P} t$.
2. (**Success**) *A proposition is always true after being successfully tested* (and so by 1 the test can be repeated): i.e., if $s \mathop {\rightarrow }\limits ^{P} t$ then $t\in P$.
3. (**Repeatability**) *A successful test can be repeated without disturbing the state*: i.e., if $s \mathop {\rightarrow }\limits ^{P} t$ then $t\mathop {\rightarrow }\limits ^{P} t$.
4. (**Redundancy**) *Repeating a successful test is a redundant action*: the same output state can be obtained by testing only once; i.e., if $s \mathop {\rightarrow }\limits ^{P} t \mathop {\rightarrow }\limits ^{P} w$ then $s\mathop {\rightarrow }\limits ^{P} w$.

The states $s\in S$ represent possible ontic states of a given system under observation. The sets $P\in {\mathcal L}$ represent propositions (about the observed system) that can be established (“known”) by tests performed by an implicit observer. Such propositions are “testable” in the weak sense given by the Testability and Success Postulates: whenever they are true they *can* be successfully tested; and, after being successfully tested they are *necessarily* true, i.e., “known” to be true for sure. Any immediate repetition of the test will leave the state unchanged and thus yield no new information. Since we assume that the observer’s knowledge about the given system is based only on observations, we can also interpret the sets in ${\mathcal L}$ as representing possible information states of the observer (or “theories”), in which case we may use letters $T\in {\mathcal L}$ to denote them. The assumption that ${\mathcal L}$ is closed under finite intersections encodes the fact that the observer can “cumulate” classical observations (i.e., if the observer’s current information state is *T* then after classically observing *P* her information state will be $T\cap P$), while closure under strongest postcondition expresses cumulation of non-classical (disturbing) observations (i.e., if the observer’s current information state is *T*, then after non-classically observing *P* her information state will be $\mathop {\rightarrow }\limits ^{P}[T]$). Closure of ${\mathcal L}$ under arbitrary intersections is a more idealized requirement, allowing the observer to form information states as infinite sets of potentially observable propositions (“theories”). Such information states can encode knowledge, beliefs or “predictions” about possible observations. Closure under weakest preconditions says that one can test (establish) whether a certain testable proposition *Q* is established by a successful test of *P* (indeed, this can be intuitively checked by first testing for *P* and then testing for *Q*)^2^.

It follows from the above assumptions that *every state has* $\mathop {\rightarrow }\limits ^{S}$-*successors and no state has* $\mathop {\rightarrow }\limits ^{\emptyset }$-*successors*: i.e., for every state $s\in S$, we have $\mathop {\rightarrow }\limits ^{S}[s]\not =\emptyset$ and $\mathop {\rightarrow }\limits ^{\emptyset }[s]=\emptyset$. Also, the notation ${\mathcal L}$ for the family of testable propositions can be justified by noticing that the above assumptions imply that $({\mathcal L}, \subseteq )$ is a *complete lattice* when ordered by set-inclusion $\subseteq$, having arbitrary intersection $\bigcap$ as its *infimum* operation.

**Testable Closure** Although in an arbitrary test frame it is possible that not all propositions are testable (i.e., that ${\mathcal L}\not ={\mathcal P}(S)$), it is nevertheless the case that *every proposition has a strongest testable consequence* *cl*(*P*): namely, the conjunction of all its testable consequences. Epistemically, *cl*(*P*) represents *the information state of the observer after learning* *P*: the conjunction of all the testable predictions based on only knowing *P*. For any proposition $\Phi \subseteq S$ (not necessarily testable), we denote by $cl(\Phi ) {:=}\bigcap \{P\in {\mathcal L}: \Phi \subseteq P\}$ the *“testable closure” of* $\Phi$. Note that *testable closure is always testable* (i.e., $cl(\Phi )\in {\mathcal L}$), *it is entailed by* $\Phi$ (i.e., $\Phi \subseteq cl (\Phi )$), and in fact *it is the strongest testable proposition entailed by* $\Phi$ (i.e., we have $cl(\Phi )\subseteq P$ for all $P\in {\mathcal L}$ s.t. $\Phi \subseteq P$).

*Revision* Given any proposition $T\subseteq S$ (typically, but not necessarily, a testable one $T\in {\mathcal L}$, thought of as a “theory” or information state) and any arbitrary proposition $\Phi \subseteq S$, we define the *revision* $T * \Phi$ *of* *T* *with* $\Phi$ by putting

$$\begin{aligned} T * \Phi \,\, {:=}\,\, \mathop {\rightarrow }\limits ^{cl(\Phi )} [T] =\{w\in S: \exists t\in P (t \mathop {\rightarrow }\limits ^{cl(\Phi )} w)\}. \end{aligned}$$

Formally, we can think of the revision operator as a result of “lifting” the test relations $\mathop {\rightarrow }\limits ^{cl(\Phi )}$ from the level of states $s\in S$ to the level of propositions (sets of states) $T\subseteq S$. In particular, when $T=\{s\}$ is a singleton, we put

$$\begin{aligned} s * \Phi \,\, {:=}\,\, \{s\} * \Phi =\{w\in S: s \mathop {\rightarrow }\limits ^{cl(\Phi )} w\}. \end{aligned}$$

It is easy to see that we always have:

$$\begin{aligned}&T\cap cl(\Phi )\not =\emptyset \hbox { implies } T * \Phi \not =\emptyset \\&T * \Phi \subseteq cl(\Phi )\\&(T * \Phi )* \Phi = T *\Phi \end{aligned}$$

**Orthogonality: impossibility of testing** We say that a state $s\in S$ is orthogonal to a proposition $\Phi \subseteq S$, and write $s\perp \Phi$, if $s * \Phi =\emptyset$. We say that proposition $T\subseteq S$ is *orthogonal* to a proposition $\Phi \subseteq S$, and write $T\perp \Phi$, if all states $s\in T$ are orthogonal to $\Phi$. It is easy to see that we have $P\perp \Phi$ iff $T * \Phi =\emptyset$. In particular, if $P\in {\mathcal L}$ is testable, then we have $s\perp P$ if the test for *P* cannot be successfully performed on *S* (i.e.,  for any *t*), and we have $T\perp P$ iff no test for *P* is successful on any state in *T*. We put

$$\begin{aligned} \sim \Phi \,{:=}\,\{s\in S: s\perp \Phi \} \end{aligned}$$

for the set of all states orthogonal to $\Phi$, and call this the *orthocomplement* of $\Phi$.

**Possibility and necessity** We say that proposition $\Phi$ is *possible* at state *s* (and write $s\in \Diamond \Phi$) if it can be successfully tested at *s*, i.e., if $s\not \perp \Phi$. So $\Diamond \Phi =\{s: s\not \perp \Phi \}=S{\setminus } \sim \Phi$ (the negation of the orthocomplement of $\Phi$) is the set of states at which $\Phi$ can be successfully tested. We say that $\Phi$ is *necessary* at *s* (and write $S\in \Box \Phi$) if its negation is impossible to test, i.e., if $s\not \perp (S{\setminus } \Phi )$. The set $\Box \Phi = S{\setminus } \Diamond (S{\setminus } \Phi ) = \{s: s\perp (S{\setminus }\Phi )\}=\{s: s * \lnot \Phi =\emptyset \}$ is the states at which $\lnot \Phi$ *cannot* be successfully tested.

**Normal states and the observer’s beliefs** A state *s* is *P*-*normal* if testing *P* may leave it undisturbed, i.e., we have $s \mathop {\rightarrow }\limits ^{P} s$. The *P*-normal states can be thought of as “typical” or default *P*-states. The set ${\mathcal N}(P){:=}\{s\in S: s \mathop {\rightarrow }\limits ^{P} s\}$ of *P*-normal worlds represents the *doxastic state* of an observer whose information state is *P*: if all the observer knows is that the observed state is in *P*, then she *believes* that it is a typical *P*-state (in ${\mathcal N}(P)$). It is easy to see that, for all information states $P, T\in {\mathcal L}$, we have:

$$\begin{aligned}&{\mathcal N} (P)\subseteq P,\\&{\mathcal N} ({\mathcal N} (P))={\mathcal N}(P),\\&P\subseteq T \, \, \hbox { implies } \,\, T * P= \mathop {\rightarrow }\limits ^{P} [T]= {\mathcal N} (P). \end{aligned}$$

### Example 1

*PDL* Tests. The “test” actions of Propositional Dynamic Logic (*PDL*) can be obtained as a special case: *classical test frames* $(S, \{\mathop {\rightarrow }\limits ^{P}\}_{{ P}\in {\mathcal L}})$, where we take ${\mathcal L} ={\mathcal P} (S)$ (i.e., *all propositions are testable*, so $cl(\Phi )=\Phi$) and $\mathop {\rightarrow }\limits ^{P}{:=} \mathop {\rightarrow }\limits ^{P?}= \{(s,s): s\in P\}$ is the *diagonal of* *P*. In other words, classical *PDL* tests $\mathop {\rightarrow }\limits ^{P?}$ are actions that can only happen in a state *s* iff *P* is true at *s*, in which case the state *s* is left unchanged. It is easy to see that *in a classical test frame, all states are normal* (${\mathcal N}(P)=P$), *revision is the same as intersection* ($T*\Phi =T\cap \Phi$) and, for every state *s*, *the revision* $s *\Phi$ *is either the singleton* $\{s\}$ *or the empty set* $\emptyset$.

**Extended Test Frames** Even in a *non-classical* test frame $(S, \{\mathop {\rightarrow }\limits ^{P}\}_{{ P}\in {\mathcal L}})$, it may still be convenient from a theoretical perspective to define the classical tests $\mathop {\rightarrow }\limits ^{P?}{:=} \{(s,s): s\in P\}$. In other words, we can consider *any* test frame as an extended structure $(S, \{\mathop {\rightarrow }\limits ^{P}, \mathop {\rightarrow }\limits ^{P?}\}_{{ P}\in {\mathcal L}})$, with classical tests $\mathop {\rightarrow }\limits ^{P?}$ (given by the diagonal of *P*) representing some kind of *idealized, non-disturbing test actions*. In this way, we can always compare within the same framework the “real” (potentially disturbing) test actions with their idealized (classical) counterparts.

**The Language of** $\varvec{DLT}$ The *dynamic logic of test* (*DLT*) has the following language:

$$\begin{aligned} \begin{array}{ccc ccc ccc ccc cc ccccc} \varphi&{:: =}&\bot |&p |&\varphi \wedge \varphi |&K\varphi |&[\varphi ?_c] \psi |&[\varphi ?_q]\varphi |&[\varphi !_c]\varphi |&[\varphi !_q]\varphi \end{array} \end{aligned}$$

where $\bot$ is a symbol for the *inconsistent proposition* (*falsum*); symbols $p\in P$ come from a given set $\Omega$ of *atomic (elementary) propositions* $\Omega$ (denoting “ontic facts”); $\varphi \wedge \psi$ is the (classical) conjunction of the two propositions; $K\varphi$ means “$\varphi$ is *known* (by the implicit agent, the “observer”); $\varphi ?_c$ is the *classical (unobserved) test* action (i.e., standard *PDL* test), that can only take place in a state if $\varphi$ is true, in which case the state is left unchanged; $[\varphi ?_q]\varphi$ is the *“dynamic” or non-classical test* (unobserved, but state-disturbing), that can only take place in a state in which $\varphi$ can *become* true, in which the state is indeed changed to any of the “closest” $\varphi$-states; $\varphi !_c$ is the *classical observation*, by which the observer learns $\varphi$ without disturbing the state); and finally, $\varphi !_q$ is the *’dynamic’ or non-classical observation*, by which the observer learns $\varphi$ by an action that changes the state to any of the “closest” $\varphi$-states. Naturally, in the quantum applications, the non-classical observations or tests will be interpreted as (observed or unobserved) *quantum* measurements.

**Testable Formulas** The *testable (or ‘dynamic’) fragment* $DLT^d$ of the language *DLT* is the set of all *DLT*-formulas that do *not* use any classical tests $\varphi ?_c$ or classical observations $\varphi !_c$. As we will see, *the formulas of the testable fragment express testable propositions*.

**Abbreviations**. We use the following abbreviations^3^:

$$\begin{aligned}&\varphi \rightarrow \psi \,\, {:=}\,\, [\varphi ?_c]\psi \,\, \hbox { for classical implication}\\&\varphi \rightsquigarrow \psi \,\, {:=}\,\, [\varphi ?_c]\psi \hbox { for dynamic (``quantum,'' or}\\&\quad \hbox { ``counterfactual'') conditional}\\&\lnot \varphi \,\, {:=}\,\, [\varphi ?_c]\bot = \varphi \rightarrow \bot \,\\&\quad \hbox {for classical negation}\\&\sim \varphi \,\, {:=}\,\, [\varphi ?_q]\bot =\varphi \rightsquigarrow \bot \\&\quad \hbox { for orthocomplement (dynamic, or ``quantum,'' negation)}\\&\top \,\, {:=}\,\, \lnot \bot =\sim \bot \,\, \hbox { for tautology }(verum )\\&\varphi \wedge \psi \,\, {:=}\,\, \lnot (\varphi \rightarrow \lnot \psi ) \hbox { for classical conjunction}\\&\varphi \vee \psi \,\, {:=}\,\, \lnot (\lnot \varphi \wedge \lnot \psi ) \hbox { for classical disjunction}\\&\varphi \sqcup \psi \,\, {:=}\,\, \sim (\sim \varphi \wedge \sim \psi ) \hbox { for dynamic (``quantum'') }\\&\quad \hbox { disjunction}\\&B^{\varphi }\psi \,\, {:=}\,\, K(\varphi \rightsquigarrow \psi ) \hbox { for }{} \textit{conditional belief}\hbox { (or ``counterfactual }\\&\quad \hbox { knowledge'')}\\&B \psi \,\, {:=}\,\, B^{\top } \psi \,\, \hbox { for }{} belief \\&\langle \alpha \rangle \psi \,\, {:=}\,\, \lnot [\alpha ] \lnot \psi \hbox { for } \textit{possibility of achieving}\, \psi \, \textit{by action}\,\, \alpha \in \\&\quad \{\varphi ?_c, \varphi ?_q,\varphi !_c, \varphi !_q\}\\&\Diamond \varphi \,\,{:=}\,\, \lnot \sim \varphi = \lnot [\varphi ?_q]\bot \hbox { for }{} \textit{possibility} \hbox { of (successful}\\&\quad \hbox { testing of) } \varphi \\&\Box \varphi \,\,{:=}\,\, \lnot \Diamond \lnot \varphi =\sim \lnot \varphi =[\lnot \varphi ?_q]\bot \hbox { for }{} \textit{necessity}\\&\quad \hbox { (=impossibility of negative testing) of }\varphi \end{aligned}$$

**Models** A *test model* $M=(S,\{\mathop {\rightarrow }\limits ^{P}\}_{{ P} \in {\mathcal L}}, \Vert \bullet \Vert )$ for our language *DLT* consists of a test frame $(S,\{\mathop {\rightarrow }\limits ^{P}\}_{{ P} \in {\mathcal L}})$ together with a valuation (or “interpretation”) map $\Vert \bullet \Vert : \Omega \rightarrow {\mathcal L}$, associating to every atomic formula $\in \Omega$ some testable property $\Vert p\Vert \in {\mathcal L}$.

**Semantics** Our semantics is really in the spirit of Dynamic Epistemic Logic, although we choose to state it in a form that is inspired from Subset Space Logic (or “topo-logic”), see Moss and Parikh (1992).^4^ We evaluate formulas, not at states, but at pairs (*s*, *T*) with $s\in T\in {\mathcal L}$, consisting of a state $s\in S$ and a factive information state $T\in {\mathcal L}$ (where “factive” means that $s\in T$). For an given “possible world” (*s*, *T*), the first component *s* represents the *ontic state* (i.e., the state of the observed system), while the second component *T* represents the *information state of the “observer”*: in other words, in world (*s*, *T*) the observes knows only that the observed state is in *T*. For a given model *M*, we define a satisfaction relation $(s,T)\models \varphi$ between such pair-worlds (*s*, *T*) and formulas $\varphi$ of *DLT*. This is equivalent to extending the interpretation map $\Vert \bullet \Vert$ from atomic formula to *all* formulas, by defining an *interpretation* $\Vert \varphi \Vert _T\subseteq S$ for each formula $\varphi$ and each information state $T\in {\mathcal L}$. The two notions are interdefinable, by putting

$$\begin{aligned}&\Vert \varphi \Vert _T \,\, {:=} \,\, \{s\in T: (s, T)\models \varphi \} \\&\quad \hbox { (and conversely, putting } (s,T)\models \varphi \hbox { iff }s\in \Vert \varphi \Vert _T). \end{aligned}$$

But it is convenient to use *both* notations in our recursive definition, as we will do below. For any information state $T\in {\mathcal L}$ and formula $\varphi$, we also use the notation

$$\begin{aligned} T * \varphi \, \, {:=}\,\, T * \Vert \varphi \Vert _T= \mathop {\rightarrow }\limits ^{cl(\Vert \varphi \Vert _T)} [T] \end{aligned}$$

Epistemically, if *T* is the observer’s current information state, then $T * \varphi$ represents her *information state after performing a successful (non-classical state-disturbing) observation of* $\varphi$. Note that, in general, *even a non-informative observation may disturb the observed state*.^5^ Since the observer is aware of this, it follows that *even a non-informative observation may change the observer’s information state*: indeed, we have

$$\begin{aligned} T*\top =T*\Vert \top \Vert _T=T * T= {\mathcal N}(T), \end{aligned}$$

which may indeed be *different* from *T*. In fact, when *T* is the observer’s information state, then $T * \top ={\mathcal N}(T)$ represents *the observer’s (default) beliefs* (given her information *T*).

For basic formulas, we put $\Vert \bot \Vert _T=\emptyset$, i.e.,

$$\begin{aligned} (s, T) \,\, \not \models \bot ; \end{aligned}$$

and $$\Vert \varphi \wedge \psi \Vert _T=\Vert \varphi \Vert _T\cap \Vert \psi \Vert _T$$, i.e.,

$$\begin{aligned} (s,T)\models \varphi \wedge \psi \,\, \hbox { iff }\,\, (s,T)\models \phi \hbox { and } (s,T)\models \psi . \end{aligned}$$

For *knowledge*, we put $s\in \Vert K\varphi \Vert _T$ iff $T\subseteq \Vert \varphi \Vert _T$, i.e.,

$$\begin{aligned} (s, T)\models K\varphi \,\, \hbox { iff }\,\, (t, T)\models \varphi \hbox { for all } t\in T. \end{aligned}$$

*Classical test* modality is nothing but classical *implication*:

$$\begin{aligned} (s, T)\models [\varphi ?_c]\psi \,\, \hbox { iff }\,\, (s,T)\models \varphi \hbox { implies } (s,T)\models \psi . \end{aligned}$$

*Classical observation* is really the standard *update* modality (“public announcement”) from Public Announcement Logic:

$$\begin{aligned} (s, T)\models [\varphi !_c]\psi \,\, \hbox { iff }\,\, (s,T)\models \varphi \hbox { implies } (s,cl(\Vert \varphi \Vert _T))\models \psi . \end{aligned}$$

For *dynamic test*, we put $\Vert [\varphi ?_q]\psi \Vert _T=\{s\in S: s * \Vert \varphi \Vert _T\subseteq \Vert \psi \Vert _T\}$, i.e.,

$$\begin{aligned} (s, T)\models [\varphi ?_q]\psi \,\, \hbox { iff }\,\, (t,T)\models \psi \hbox { for all } t\in s *\Vert \varphi \Vert _T. \end{aligned}$$

For *dynamic observation*, we put $\Vert [\varphi !_q]\psi =\{s\in S: s * \Vert \varphi \Vert _T\subseteq \Vert \psi \Vert _{T * \Vert \varphi \Vert _T}\}$, i.e.,

$$\begin{aligned} (s, T)\models [\varphi !_q]\psi \,\, \hbox { iff }\,\, (t,T * \Vert \varphi \Vert _T)\models \psi \hbox { for all } t\in s *\Vert \varphi \Vert _T. \end{aligned}$$

It is essential to notice that *all our connectives, with the exception of conjunction, are in fact Kripke modalities* (although *not* for ontic accessibility relations $R\subseteq S\times S$ between states, but) *for accessibility relations between “worlds” as pairs* (*x*, *T*): indeed, given any binary relation $\mathop {\longrightarrow }\limits ^{R}$ on world-pairs (*s*, *T*), we can introduce a Kripke modality by putting

$$\begin{aligned}&(s,T)\models [\mathop {\longrightarrow }\limits ^{R}]\psi \,\, \hbox { iff }\,\, (s', T')\models \psi \hbox { for all } (s', T') \\&\qquad \hbox { with } (s,T)\mathop {\longrightarrow }\limits ^{R} (s', T'). \end{aligned}$$

Given this, we can easily see that our connectives above are Kripke modalities for the accessibility relations given by:

$$\begin{aligned}&(s, T) \mathop {\longrightarrow }\limits ^{K} (s',T){,} \hbox { for all } s'\in T,\\&(s, T) \mathop {\longrightarrow }\limits ^{\varphi ?_c} (s,T){,} \hbox { for all } s\in \Vert \varphi \Vert _T,\\&(s, T) \mathop {\longrightarrow }\limits ^{\varphi ?_q} (s',T){,} \hbox { for all } s'\in s * \Vert \varphi \Vert _T,\\&(s, T) \mathop {\longrightarrow }\limits ^{\varphi !_c} (s,cl(\Vert \varphi \Vert _T)){,} \hbox { for } s\in \Vert \varphi \Vert _T,\\&(s, T) \mathop {\longrightarrow }\limits ^{\varphi !_q} (s',T * \Vert \varphi \Vert _T){,} \hbox { for all } s'\in s * \Vert \varphi \Vert _T. \end{aligned}$$

### Proposition 1

Given a model *M* and an information state $T\in {\mathcal L}$, *the interpretation in* *T* *of any testable formula is a testable proposition*: i.e., if $\varphi$ is a formula of the testable fragment $DLT^d$ then $\Vert \varphi \Vert _T\in {\mathcal L}$.

A formula $\varphi$ is *valid on a test model* $M=(S,\{\mathop {\rightarrow }\limits ^{P}\}_{{ P} \in {\mathcal L}}, \Vert \bullet \Vert )$ if $\Vert \varphi \Vert _T=S$ for all information states $T\in {\mathcal L}$; i.e., if $\varphi$ it is true at all world-pairs (*s*, *T*) with $s\in T\in {\mathcal L}$. The formula $\varphi$ is *valid on a test frame* $(S, \{\mathop {\rightarrow }\limits ^{P}\}_{{ P} \in {\mathcal L}})$ if it is valid on all models on this frame (regardless of valuation). Finally, it is *valid on a class* *K* *of frames* if it is valid on all frames in the class *K*.

### Proposition 2

All our modalities *K*, $[\varphi ?_c]$, $[\varphi ?_q]$, $[\varphi !_c]$, $[\varphi !_q]$ are normal, i.e., validate Kripke’s axiom (e.g., $K(\varphi \rightarrow \theta )\rightarrow (K\varphi \rightarrow K\theta )$) and Necessitation Rule (e.g., if $\varphi$ is valid, then $K\varphi$ is valid).

### Proposition 3

For all formulas $\varphi$ and $\theta$, the following formulas are valid on the class of all test frames:

$$\begin{aligned}&K\varphi \rightarrow \varphi \\&K\varphi \rightarrow K K\varphi \\&\lnot K\varphi \rightarrow K\lnot K\varphi \\&K\varphi \rightarrow (\theta \rightsquigarrow \varphi )\\&K\varphi \rightarrow B^{\theta } \varphi \\&B^{\varphi } \theta \rightarrow K B^{\varphi }\theta \\&\lnot B^{\varphi } \theta \rightarrow K \lnot B^{\varphi }\theta \\&\langle \varphi ?_c\rangle \top \leftrightarrow \varphi \\&\langle \varphi ?_q\rangle \top \leftrightarrow \Diamond \varphi \\&\langle \varphi !_c \rangle \top \leftrightarrow \varphi \\&\langle \varphi ?_q\rangle \top \leftrightarrow \Diamond \varphi \end{aligned}$$

The last four validities express the “ontic preconditions” of our actions: the classical test or observation of $\varphi$ can succeed iff $\varphi$ is true in the initial state (before the action), while dynamic test or observation of $\varphi$ can succeed iff $\Diamond \varphi$ is true in the initial state (before the action).

### Proposition 4

For all *testable* formulas $\varphi$ and all atomic formulas $p\in \Omega$, the following formulas are valid on the class of all test frames:

$$\begin{aligned}&B^{\varphi } \varphi \\&K\varphi \rightarrow (\lnot \varphi \rightsquigarrow \bot )\\&K\varphi \rightarrow B^{\lnot \varphi } \bot \\&[\varphi ?_q] \varphi \,\,\, \hbox { , i.e., }\varphi \rightsquigarrow \varphi \\&[p!_c] K p\\&[p!_q] K p \end{aligned}$$

The last three validities give us the ontic postconditions of these actions, while the ontic postcondition of classical test is trivial $[\varphi ?_c]\varphi$ holds for *arbitrary* (not necessarily testable) $\varphi$.

## Examples

Besides classical *PDL* tests, we show now how our framework subsumes other examples, including plausibility models for belief revision, sphere models for counterfactual reasoning and quantum test frames.

### Example 2

*CDL* *Frames* Conditional Doxastic Logic (*CDL*) can be obtained as another special case: any *plausibility frame* $(S,\le )$ generates a *conditional doxastic frame* $(S, \{\mathop {\rightarrow }\limits ^{P}\}_{{ P}\in {\mathcal L}})$, by taking ${\mathcal L} ={\mathcal P} (S)$ (i.e., *all propositions are testable*, so $cl(\Phi )=\Phi$), and defining *conditional doxastic accessibility relations* $\mathop {\rightarrow }\limits ^{P}:= \{(s,t): s\in S, t\in Min_{\le } P\}$, where $Min_{\le } P=\{s\in P: s\le t \hbox { for every } t\in P\}$ is the set of minimal (“most plausible”) *P*-states. It is easy to see that in *CDL* frames, we have ${\mathcal N}(P)=Min_{\le } P$ and $T * \Phi =Min_{\le } \Phi$. It is also easy to check that conditional beliefs $B^{\varphi }\psi$, knowledge $K\varphi$ and classical update $[\varphi !_c]\psi$, as defined in this *CDL* test frame coincide with the corresponding notions as defined in the given plausibility frame. Moreover, in *CDL* frames *conditional belief* $B^{\varphi }\psi$ *and dynamic conditional* $\varphi \rightsquigarrow \psi$ *are the same notion*. As for dynamic observation $\varphi !_q$, this is not a realistic action in the doxastic context: it corresponds to observing (or even performing) an action by which all the observer’s beliefs are miraculously fulfilled.^6^ Also, in *CDL* frames, *knowledge coincides with “necessity”* (i.e., $\Box \varphi$ is the same as $K\varphi$), and *orthocomplement is the same as knowledge of falsehood* (i.e., $\sim \varphi$ is equivalent to $K\lnot \varphi$).

### Example 3

*Models for Counterfactual Conditionals* A *Lewis frame* for counterfactuals is a structure $(S, \le _s)_{s\in S}$ consisting of a state of states *S* together with a family of *similarity relations* $\le _s\subseteq S\times S$, with the following properties: each $\le _s$ is a (non-empty) well-founded preorder, it is “total on its domain” $dom(\le _s):=\{s\in S: s\le t \hbox { for some } t\in S\}$ (i.e., $w,t\in dom(\le _s)$ implies that we have either $w\le _s t$ or $t\le _s w$), and it is “strongly centered” (i.e., we have $w\le _s s$ iff $w=s$). Similarity frames are the standard models for counterfactual conditionals (equivalent to Lewis “sphere models”). They can be structured as test frames by taking ${\mathcal L} ={\mathcal P} (S)$ (i.e., *all propositions are testable*, so $cl(\Phi )=\Phi$), and defining *counterfactual conditional relations* $\mathop {\rightarrow }\limits ^{P}{:=} \{(s,t): s\in S, t\in Min_{\le _s} P\}$, where $Min_{\le _s} P=\{w\in P: w\le _s t \hbox { for every } t\in P\}$ is the set of $\le _s$-minimal *P*-states (i.e., the *P*-states that “are most similar,” or “closest,” to *s*). *In Lewis frames, all states are normal* (i.e., ${\mathcal N}(P)=P$). Using Lewis’ terminology, we can think of a counterfactual test of *P* as mentally performing a “small miracle” that changes the world in a minimal way to make *P* true (if at all possible). In Lewis models, the dynamic conditional $\varphi \rightsquigarrow \psi$ is exactly Lewis’ counterfactual conditional, and our necessity operator $\Box$ coincides with Lewis’ definition of necessity, while orthocomplement $\sim \varphi$ is the same as Lewis impossibility $\lnot \Box \varphi$.

**Table 1:** An overview of these similarities and differences

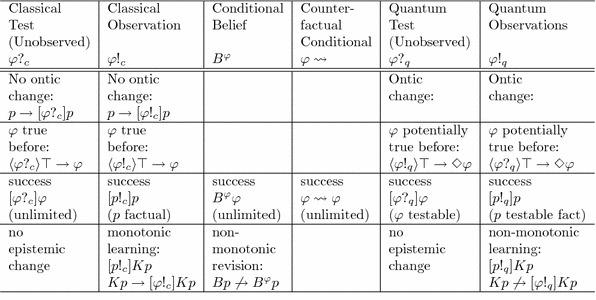	

**Epistemic Counterfactual Logic** One should note that our setting adds to counterfactual logic an “observer” and her knowledge *K*, so we obtain an *epistemic counterfactual logic*. Note that in Lewis frames, *(default) “belief”* $B\varphi$ *(as defined above) is the same as knowledge* $K\varphi$, and *“conditional belief”* $B^{\varphi }\psi$ *is the same as knowledge of the counterfactual* $K(\varphi \rightsquigarrow \psi )$. In other words, this observer is “un-opinionated”: she never goes beyond what she knows. So this is an realistic setting only for an epistemic (rather than a doxastic) extension of counterfactual logic.

### Example 4

**Quantum Test Frames** A Hilbert space *H* can be structured as a *quantum test frame*, by taking as set of states *S* the set of all “rays” (one-dimensional subspaces) of *H*, taking the family ${\mathcal L}$ of testable properties to consist of all (subsets of *S* corresponding to) closed linear subspaces *P* of *H*, and taking $\mathop {\rightarrow }\limits ^{P}$ to be the (partial function induced on *S* by) the *projector* $Pr_P$ onto the linear subspace *P*. Then $cl(\Phi )$ is the closed linear subspace generated by $\Phi$. In a quantum frame, *all states are normal* (${\mathcal N}(P)=P$), since *quantum* *P*-*tests do not disturb any* *P*-state (i.e., if $s\in P\in {\mathcal L}$, then we have $s \mathop {\rightarrow }\limits ^{P} t$ iff $s=t$).

**Quantum Dynamic Epistemic Logic** Once again, note that our setting adds to quantum dynamic logic an “observer” and her knowledge *K*, as well as information updates corresponding to (classical and quantum) observations. So, even when restricted to quantum test frames, our logic *DLT* we obtain a *quantum version of Dynamic Epistemic Logic* (*DEL*), which can be used to talk about the informational effects of both classical and quantum measurements. The *“purely quantum” DEL* is the testable fragment of this logic (obtained by eliminating classical tests and classical observations), whose formulas can always be interpreted as “experimental propositions” describing testable properties of a quantum-informational system. Note that (like the case of Lewis frames), *in a quantum test frame “belief”* $B\varphi$ *(as defined above) is the same as knowledge* $K\varphi$, and *“conditional belief”* $B^{\varphi }\psi$ *is the same as knowledge of the quantum implication* $K(\varphi \rightsquigarrow \psi )$. So our quantum observer is also “un-opinionated”: her beliefs are only based on the available information.

## Comparing various types of conditionals

Our unified framework allows us to easily compare the different types of conditionals and forms of learning mentioned above. Quantum test operators $\varphi ?_q$ and quantum observations $\varphi !_q$ share some common features with classical *PDL* tests $\varphi ?_c$, classical observations $\varphi !_c$, doxastic conditionals $B^{\varphi }$ and counterfactual conditionals $\varphi \rightsquigarrow$. All these operators can be thought as weakest preconditions (Kripke modalities) for abstract “test” actions, by which a proposition is tested on a given state. Each of them satisfies some version of the Success postulate. But there are some differences as well. Classical tests and classical observations preserve the ontic facts (i.e., the non-epistemic features of the system, that depend only on its state, stay the same), but quantum tests and quantum observations may induce ontic changes: “in quantum mechanics, epistemic actions have ontic side effects.” (As for doxastic and counterfactual conditionals, they are purely hypothetical, imaginary transformations, rather than real actions, so the question of ontic changes is meaningless for them.) Classical tests $\varphi ?_c$ and classical observations $\varphi !_q$ can only happen if the tested proposition was true (*before* the action), so they have $\varphi$ as their *precondition*; in contrast, successful quantum tests $\varphi ?_q$ and quantum observations $\varphi !_q$ can happen in any state *s* in which $\varphi$ was *potentially true* (i.e., $s\not \perp \varphi$), so their precondition is the possibility statement $\Diamond \varphi$. (Once again, the question is meaningless for the other conditionals.) The various types of test satisfy different forms of Success, some in unrestricted form (valid for all propositions), and some only with restrictions (e.g., only for purely factual propositions *p* describing ontic facts, or only for testable propositions $\varphi$, or only for testable facts *p*): see Table 1 for a detailed overview of these different forms of Success. From an *informational* perspective, there are also a number of differences: unobserved tests (classical or quantum) involve no informational dynamics (since they do not affect the observing agent), and the question is again meaningless for counterfactual conditionals (since they do not actually concern the observer). In contrast, *new knowledge or new beliefs are acquired by the other forms of conditioning* (only hypothetically in the case of doxastic conditionals, or actually acquired via classical and quantum observations). Finally, *the informational dynamics is monotonic in the case of classical observations* (once a fact is learned, it continues to be known after new observations), but it is *non-monotonic in the case of belief revision* (since old beliefs can be overturned) *and of quantum observations* (since old knowledge can become obsolete because of the state change induced by new observations, and the results of old measurements can be undone by new quantum measurements).

Let us stress that, while quantum tests and quantum observations do share some formal features with purely epistemic/doxastic actions in Belief Revision theory and update operators in Dynamic Epistemic Logic, we are *not* claiming to reduce or explain quantum behavior to a purely informational phenomenon. The quantum realm is characterized by the *erosion of the sharp classical separation between “ontic” and “epistemic”* (Baltag and Smets 2008a). This is a major difference from the logic of classical observes and belief revision. *In a quantum universe, there are no “purely epistemic” actions*. So it would be meaningless to attempt to reduce quantum weirdness to pure information, or to “explain” quantum superposition, entanglement, correlations, etc. as nothing but epistemic effects, due exclusively to the observer’s uncertainty and lack of information.

On the contrary, a dynamic quantum perspective aims to explain knowledge, information gathering (observations, etc.), as well as information processing (combining information, thinking, reasoning counterfactually, interpreting the phenomena), in terms of actual interactions and correlations between actually existing systems. The observer lives in the world, and her learning actions are real physical events, which have real consequences. In this sense, Marx’ famous quote on the imperative to change the world^7^ is a red herring. Whether we want it or not, we are all changing the world every time we interpret it.

## Footnotes

1 This means that $\le$ is a reflexive and transitive relation, with the property that every two states $s,t\in S$ are comparable (i.e., either $s\le t$ or $t\le s$) and for every non-empty subset $P\subseteq S$ the set of its minimal elements $Min_{\le } P$ is also non-empty.

2 This is *not* necessarily the same as closure of test actions under sequential composition! The sequential composition of successful tests $\mathop {\rightarrow }\limits ^{P} ; \mathop {\rightarrow }\limits ^{Q}$ establishes indeed the proposition that “*Q* is established by successfully testing *P*,” so that the sequential composition is *epistemically equivalent* to the single test of this proposition. But the two might be *non-equivalent with respect to their ontic effects*: the sequential composition may disturb the state of the observed system in a way that is the *different* from the disturbance induced by directly testing the above-mentioned proposition. Indeed, this is exactly what happens in the quantum case!

3 In fact, the classical conjunction $\phi \wedge \psi$ is also definable as an abbreviation using classical tests and the falsum $\bot$, but we chose to considerate it as a primitive symbol, because of its importance for testability.

4 In the single-agent case, every version of Dynamic Epistemic Logic, Public Announcement Logic etc can be reformulated in this way.

5 However, this phenomenon does *not* happen in quantum mechanics. Testing quantum properties that are already known does not change a quantum system.

6 Cf. Frank Lloid Wright: “The thing always happens that you really believe in; and the belief in a thing makes it happen.”

7 “The philosophers have only interpreted the world, in various ways. The point, however, is to change it.” (Karl Marx)

## References

[CR1] Alchourrón C, Gärdenfors P, Makinson D (1985). On the logic of theory change: partial meet contraction and revision functions. J Symbol Logic.

[CR2] Baltag A, Smets S (2005). Complete axiomatizations for quantum actions. Int J Theor Phys.

[CR3] Baltag A, Smets S (2006a) Conditional doxastic models: a qualitative approach to dynamic belief revision. Electron Notes Theor Comput Sci 165:5–21

[CR4] Baltag A, Smets S (2006b) LQP: the dynamic logic of quantum information. Math Struct Comput Sci 16(3):491–525

[CR5] Baltag A, Smets S (2008a) A dynamic-logical perspective on quantum behavior. Stud Log 89:185–209

[CR6] Baltag A, Smets S (2008b) A qualitative theory of dynamic interactive belief revision. In: Bonanno G, van der Hoek W, Woolridge M (eds) Texts in logic and games, vol 3. Amsterdam University Press, Amsterdam, pp 9–58

[CR7] Birkhoff G, von Neumann J (1936). The logic of quantum mechanics. Annal Math.

[CR8] Coecke B, Smets S (2004). The Sasaki Hook is not a [static] implicative connective but induces a backward [in time] dynamic one that assigns causes of truth. Int J Theor Phys.

[CR9] Hardegree GM (1975). Stalnaker conditionals and quantum logic. J Philos Log.

[CR10] Hardegree GM (1979) The logico-algebraic approach to quantum mechanics, vol 2, Chap: The conditional in abstract and concrete quantum logic. D. Reidel Pub., Dordrecht

[CR11] Harel D, Kozen D, Tiuryn J (2000). Dynamic logic (foundations of computing).

[CR12] Kalmbach G (1983). Orthomodular lattices.

[CR13] Levitt SD, List JA (2009) Was there really a Hawthorne effect at the Hawthorne plant? an analysis of the original illumination experiments. Technical report, National Bureau of Economic Research. Working Paper no 15015 May

[CR14] Marsen EL, Herman L, Piziak R (1981). Implication connectives in orthomodular lattices. Notre Dame J Formal Log.

[CR15] Moore GE (1942) A reply to my critics. In: Schilpp PA (ed) The Philosophy of G.E. Moore, volume 4 of The Library of Living Philosophers. Northwestern University, Evanston, pp 535–677

[CR16] Moss LS, Parikh R (1992) Topological Reasoning and The Logic of Knowledge. In Moses Y (ed) Proceedings of the 4th Conference on Theoretical Aspecats of Reasoning about Knowledge (TARK 1992). Morgan Kaufmann, San Francisco, CA, pp 95–105

[CR17] Plaza JA (1989) Logics of public communications. In: Emrich ML, Pfeifer MS, Hadzikadic M, Ras ZW (eds) Proceedings of the 4th international symposium on methodologies for intelligent systems, pp 201–216

[CR18] Smets S (2001). On causation and a counterfactual in quantum logic: the Sasaki Hook. Log Anal.

[CR19] van Benthem J (1996) Exploring logical dynamics. CSLI Publications, Stanford

[CR20] van Benthem J (2011) Logical dynamics of information and interaction. Cambridge University Press, Cambridge

